# Identification of CAPG as a potential prognostic biomarker associated with immune cell infiltration and ferroptosis in uterine corpus endometrial carcinoma

**DOI:** 10.3389/fendo.2024.1452219

**Published:** 2024-11-12

**Authors:** Junwei Liu, Weiqiang Zhu, Lingjin Xia, Qianxi Zhu, Yanyan Mao, Yupei Shen, Min Li, Zhaofeng Zhang, Jing Du

**Affiliations:** ^1^ School of Pharmacy, Xinxiang Medical University, Xinxiang, Henan, China; ^2^ Shanghai-Ministry of Science and Technology Key Laboratory of Health and Disease Genomics, National Health Commission Key Lab of Reproduction Regulation, Shanghai Institute for Biomedical and Pharmaceutical Technologies, School of Pharmacy, Fudan University, Shanghai, China

**Keywords:** uterine corpus endometrial carcinoma, CAPG, immune status, ferroptosis, biomarker

## Abstract

**Introduction:**

Capping actin protein, gelsolin-like (CAPG) is a potential therapeutic target in various cancers. However, the potential immunotherapeutic effects and prognostic value of CAPG in uterine corpus endometrial carcinoma (UCEC) remain unclear.

**Methods:**

The characterization, methylation effects, prognostic value, targeted miRNAs of CAPG, and the correlation of CAPG with immune cell infiltration and ferroptosis in UCEC were investigated using multiple public databases and online tools. Furtherly, we explored the potential physiological function of CAPG using EdU and Transwell migration assays, identified the cell localization and expression of CAPG and GPX4 by immunofluorescence, and detected the intracellular Fe^2+^ levels using a FerroOrange fluorescent probe in Ishikawa cells. Additionally, the OncoPredict package was used to analyze the potential chemotherapeutic drugs for UCEC.

**Results:**

CAPG showed generally high expression in tumor group. The overall survival rate of the high-risk group was significantly lower than that of the low-risk group. Enrichment analysis indicated that CAPG is involved in immune-related pathways and is closely associated with the tumor microenvironment. CAPG expression levels were affected by abnormal DNA methylation and/or targeted miRNAs, infiltration levels and marker genes of various immune cells, thereby impacting immune response, ferroptosis, and patient prognosis. Ferroptosis analysis indicated that ALOX5 and VLDLR were the top CAPG-related ferroptosis markers; glutathione metabolism levels in tumor group were generally high, and decitabine was a ferroptosis inducer. CAPG-siRNA suppressed the cell proliferation and invasion, and markedly elevated the expression levels of immune-related genes IL8, TNF, TLR4 and the intracellular Fe^2+^ levels. CAPG co-located with GPX4 in nucleus and co-regulated ferroptosis and metabolism in Ishikawa cells. Moreover, four chemotherapy drugs showed better sensitivity to UCEC patients in the low-risk cohort.

**Conclusions:**

CAPG may serve as a potential biomarker of UCEC owing to its role in modulating the immune response and ferroptosis, providing novel perspectives for combined immunotherapy of UCEC.

## Graphical Abstract

In UCEC, abnormal DNA methylation and/or targeted miRNAs expression may alter the expression of CAPG, thereby impacting immune response, ferroptosis, and patient prognosis. CAPG may affect TME by regulating the immune-related genes (such as IL8, TNF, TLR4) in infiltrating immune cells. CAPG not only regulates the proliferation and invasion of Ishikawa cells but also co-locates with GPX4 in nucleus and co-regulates ferroptosis and metabolism by combining with ALOX5, VLDLR markers and inducer decitabine. The changes of ferroptosis and TME co-modulate antitumor immunity in UCEC patients, while CAPG-related chemotherapeutic agents (paclitaxel, epirubicin, docetaxel, and cyclophosphamide) for UCEC patients show better sensitivity in the low-risk expression cohort.

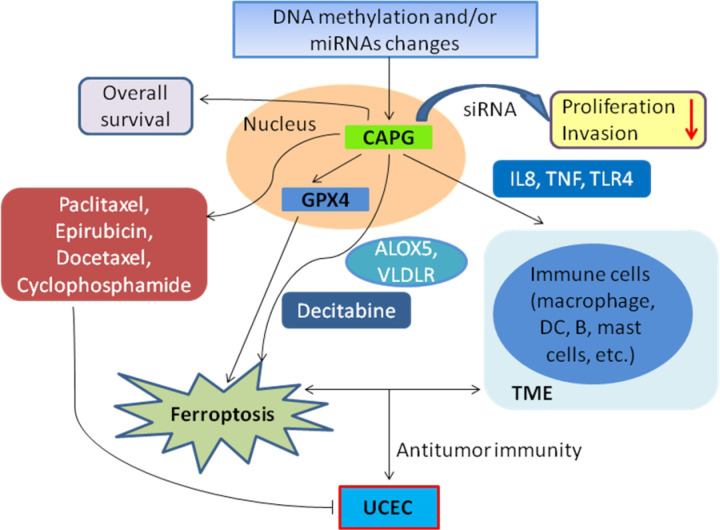

## Introduction

Uterine corpus endometrial carcinoma (UCEC) is a prevailing gynecological malignancy, with an escalation in global incidence and mortality rates. Additionally, there is a trend of this cancer occurring at a younger age (1, 2). Risk factors contributing to UCEC development include prolonged uncontested estrogen exposure, advanced age, and obesity (1, 3). Currently, the treatment strategies for UCEC beyond surgery, radiotherapy, chemotherapy, and hormone therapy remain limited (4, 5). In general, early screening and intervention have the potential to greatly decrease the incidence, recurrence, and mortality of UCEC; however, patients in advanced stages often exhibit limited response to conventional treatment, with 5-year survival rates as low as 17 percent (6). Immunotherapy, as an emerging medical therapy, has evolved as a substitute or adjunct therapeutic option to conventional radiation and chemotherapy and may hold promise for certain patient cohorts. Furthermore, combinations of different treatments can result in better outcomes (3, 7). Consequently, there is an urgent need to delve deeper into the underlying pathogenesis and effective treatment strategies for UCEC. Such endeavors have the potential to open new avenues for enhancing patient outcomes and refining UCEC management.

Tumor microenvironment (TME), which is characterized by hypoxia, acidity, and nutrient deprivation, plays a pivotal role in tumorigenesis, progression, metastasis, and therapeutic interventions. Within the TME, an intricate interplay of cells and molecules, encompassing immune cells, stromal cells, cytokines, and chemokines, prevails. This harmonious interplay jointly regulates the phenotype, anti-tumor immunity, and therapeutic response in malignant tumors (8–10). The TME has garnered considerable attention as a therapeutic target for tumors in clinical research (11).

Ferroptosis is a distinct mode of cell death driven by the accumulation of iron-dependent lipid hydroperoxides that intertwine with metabolism, REDOX biology, and pathological processes (12). However, ferroptosis in the TME not only plays a pivotal role in the regulation of infiltrating immune cells but also plays a novel role in the crosstalk between tumor cells and immune cells, which illuminates new avenues in tumor immunotherapy (13, 14). Additionally, immunotherapy-activated CD8+ T cells can down-regulate the expression of SLC3A2 and SLC7A11 by releasing interferon-γ (IFNγ), and increase the specific lipid peroxidation and ferroptosis of tumor cells, thereby enhancing the anti-tumor efficacy of immunotherapy, indicating that ferroptosis and immunotherapy have a positive feedback and kill cancer cells together (15). In recent years, the development of innovative therapies such as ferroptosis induction and immunotherapy has made significant advancements in the treatment of UCEC.

The capping actin protein, gelsolin-like (*CAPG*), also known as Macrophage Capping Protein, regulates cell motility by remodeling actin filaments, which participate in cell migration and invasion in several types of cancers (16). *CAPG* has been reported as a potential prognostic biomarker and potential clinicopathological predictor of various cancers (17). Studies on the status of natural killer (NK) cells in TME and NK heterogeneity have shown that NK cell population preferentially expressing CAPG is closely related to tumor metastasis, which is crucial for the understanding of cancer immunotherapy (18). In addition, the CAPG protein plays an important role in the molecular communication between the embryo and uterine endometrium and in the diagnosis of endometrial cancer (16, 19). *CAPG* promotes the proliferation of colorectal cancer cells by inhibiting ferroptosis (20), and ferroptosis activation may be a potential therapeutic strategy in hepatocellular carcinoma patients with high *CAPG* expression (21). Although diverse roles of *CAPG* in numerous contexts have been elucidated, its precise role in UCEC remains uncertain.

Herein, we investigated the characterization of *CAPG* and the correlation between *CAPG* and immune cell infiltration and ferroptosis in UCEC using patient data from various public databases and online tools and explored the potential physiological function of *CAPG* in Ishikawa cells through EdU and Transwell migration assays. Moreover, *CAPG*-related drug sensitivity and ferroptotic metabolism were analyzed to determine the basis of UCEC pathogenesis and treatment. These findings showed that targeting *CAPG* may be a promising therapeutic approach for the ferroptosis-induction therapy and/or combination immunotherapy for UCEC.

## Materials and methods

### Single-cell transcriptomic data analysis

The single-cell dataset (PRJNA650549) of endometrial tissues was downloaded from the Sequence Read Archive (SRA, https://www.ncbi.nlm.nih.gov/sra) with the author’s permission and comprised single-cell transcriptomes of endometrial tissues from five tumor tissues and three para-tumor tissues (22). Cells with total mitochondrial gene expression >5% and fewer than 200 genes per cell were removed after Cell Ranger software (version 2.2.0) processed the readings from each sample. We transformed the resulting matrix into a Seurat object and used harmony to merge all the Seurat objects for individual samples into one combined object (23). Uniform Manifold Approximation and Projection was used to visualize the dataset in two-dimensional space, and the cell type was tagged according to the literature or a well-known cell marker. Finally, using the “FindAllMarkers” tool, genes with *P* < 0.05 and |log_2_FC| > 0.8 were identified as differentially expressed genes from the comparison of tumors and para tumor tissues.

Furthermore, to investigate the effects of ferroptosis at the single-cell level, we calculated ferroptosis scores for both UCEC patients and healthy subjects using AddModuleScore, and analyzed the differences in ferroptosis metabolism between the two groups using scMetabolism.

### Characterization of *CAPG*

The RNA-seq information and matched clinical data of 545 subjects (including 11 controls and 532 UCEC patients with complete clinical data) were downloaded from The Cancer Genome Atlas (TCGA) database, which is open to the public and does not require approval from the local ethics committee. Patients without complete survival data were excluded. Tumor Immune Estimation Resource (TIMER) is also a public web server which encompasses 10,897 samples across 32 cancer types from TCGA database (24). The expression levels of *CAPG* in different cancer types were analyzed using the TIMER 2.0 database (https://cistrome.shinyapps.io/timer/). TCGA Gene analysis in University of ALabama at Birmingham CANcer (UALCAN) (http://ualcan.path.uab.edu/) was performed to identify *CAPG* expression in UCEC based on different categories. Immunohistochemistry for CAPG in endometrial carcinoma and adjacent tissues was obtained from the Human Protein Atlas (https://www.proteinatlas.org/).

### Survival analysis

To examine the correlation between the expression level of *CAPG* and the prognosis of UCEC patients, we categorized the patients into high-risk (high expression) and low-risk (low expression) groups determined by the average expression level of *CAPG*. Kaplan–Meier (KM) analysis was performed to compare the overall survival (OS) between the high- and low-expression cohorts using Kaplan–Meier Plotter (http://www.kmplot.com/). Moreover, prospective prognostic indicators were identified using univariate Cox analysis, and multivariate Cox analysis was used to identify independent risk factors for UCEC.

### *CAPG* promoter methylation analysis

The *CAPG* promoter region was analyzed using the Ensembl database (http://asia.ensembl.org/index.html) and CpG islands were predicted using MethPrimer 2.0 (http://www.urogene.org/methprimer2/). UALCAN was employed to analyze the *CAPG* promoter methylation levels in UCEC. The transcription factors of the target *CAPG* CpG islands were predicted using the JASPAR database (https://jaspar.genereg.net/), and potential transcription factors were first obtained from the UCSC database (http://genome.ucsc.edu/).

### Identification of low expressed miRNAs targeting *CAPG*

miRNAs that target the *CAPG* gene were predicted using the miRWalk, TargetScan, and TarBase v.8 databases. The expression and survival curves of miRNAs in UCEC were analyzed separately using the starBase (https://starbase.sysu.edu.cn/) and Kaplan–Meier Plotter (http://www.kmplot.com/) databases.

### Enrichment analysis

*CAPG*-related genes were obtained using TCGA database, and the top 300 genes with the most positive correlation with *CAPG* were screened based on the correlation score for enrichment analysis to reflect the functional roles of *CAPG*. Gene Ontology (GO) and Kyoto Encyclopedia of Genes and Genomes (KEGG) analyses were performed using the KEGG Orthology-Based Annotation System (KOBAS version 3.0) (http://kobas.cbi.pku.edu.cn).

### Immune characteristics of *CAPG*

To investigate whether *CAPG* expression was related to the tumor immune microenvironment, we first estimated the content of stromal and immune cells in tumor samples using the ESTIMATE algorithm (25). The CIBERSORT algorithm was used to assess the infiltration levels of 22-types of immune cells within both the tumor and normal groups. The correlation between *CAPG* expression and immune cell infiltration was analyzed using SPSS software. Furthermore, the scores of 28-types of immune cells were computed for both the high- and low-risk groups by employing single sample gene set enrichment analysis (ssGSEA) algorithm via the “GSVA” package of the R software.

### Chemotherapy for UCEC patients

The R package “oncoPredict” was used to assess the half-maximum inhibitory concentration (IC_50_) of several common chemotherapeutic drugs (paclitaxel, epirubicin, docetaxel, gemcitabine, topotecan, cisplatin, and cyclophosphamide) in UCEC patients. Based on the differential expression levels of *CAPG*, the sensitivity of UCEC patients to chemotherapeutic drugs was identified in low- and high-risk cohorts.

### Ferroptosis analysis

Ferroptosis-related inducers and inhibitors were downloaded from FerrDb V2 database (http://www.zhounan.org/ferrdb/current/); among them, *CAPG*-related drug sensitivity was analyzed using Cancer Therapeutics Response Portal V2 (https://portals.broadinstitute.org/ctrp/) to identify UCEC dependencies with small molecules.

### Cell culture and transfection

Human UCEC cell line Ishikawa (RRID: CVCL_2529), derived from a 39-year-old woman with endometrial adenocarcinoma, is epithelial-like, adherent growth cells that express estrogen and progesterone receptors. Ishikawa cells were obtained from the Cell Bank of Fuheng Biology (code number FH0305), Shanghai, China and were cultured at 37°C in a 5% CO_2_ incubator using Dulbecco’s modified eagle medium (DMEM) (Gibco, Grand Island, NY, USA) supplemented with 10% fetal bovine serum (FBS) (Gibco) and 1% penicillin/streptomycin (Gibco). Cell transfection was conducted using the Lipofectamine 2000 Reagent (Invitrogen) according to the manufacturer’s protocol. Cells were seeded into six-well plates for 24 h and then transfected with negative control (NC), *CAPG* siRNA (100nM, Santa Cruz Biotechnology), erastin, and ferrostatin (Merck KGaA, Germany). All experiments were performed with mycoplasma-free cells. The cell line has been authenticated using STR (or SNP) profiling within the last three years.

### RNA isolation and qRT-PCR assay

Total RNA was extracted from Ishikawa cells using TRIzol reagent (Invitrogen, Carlsbad, CA, USA). And the RNA quality (Absorbance_260/280_ 1.8–2.1) and concentration were examined t using NanoDrop 2000 (Wilmington, DE, USA). Single-stranded cDNA was synthesized using a Reverse Transcription Kit (Takara, Tokyo, Japan). We conducted quantitative real-time polymerase chain reaction (qRT-PCR) analysis on a LightCycler^®^ 480II Real-Time PCR System (Roche, Basel, Switzerland) with a SYBR-Green PCR master mix kit. Relative expression levels were analyzed using the 2^–ΔΔCt^ method and normalized to the levels of GAPDH. The primer sequences are shown in **Supplementary Table 1**.

### EdU assay

DNA synthesis (Cell proliferation) was assessed using EdU (5-ethynyl-2’-deoxyuridine) detection kit (Beyotime Biotechnology, China). Briefly, 1 × 10^5^ cells/well were seeded into 24-well plates and transfected with si-*CAPG* or NC. After incubation for 20h at 37°C, each well was added with 10 μM EdU reagent was added to each well; the plates were incubated for another 2 h and then photographed using a fluorescence microscope (Olympus, Japan). Finally, the EdU-positive rate of the cells was evaluated using ImageJ (v1.4.3) software.

### Transwell migration assay

The cell invasion assay was carried out as previously described (26). Briefly, 8 × 10^4^ cells/well were seeded in the upper chamber with serum-free medium, and DMEM with 25% FBS was added to the lower chamber for chemotaxis. After being cultured for 24 h, the cells that had migrated were fixed and subjected to staining using a solution of 0.5% crystal violet. Three fields were randomly captured, photographed and counted under a microscope.

### Immunofluorescence

Ishikawa cells were washed thrice with phosphate buffered-saline, fixed with icecold 4% paraformaldehyde for 15 min, and permeabilized in 0.3% Triton X-100 for 10 min at room temperature. Cells were incubated overnight at 4°C with anti-CAPG and anti-GPX1 (Beyotime, Shanghai, China) and stained with corresponding secondary antibody for 1h the next day. The nuclei were stained using 4′,6-diamidino-2-phenylindole (DAPI). Images were captured using a fluorescence microscope.

### Measurement of intracellular Fe^2+^

Intracellular Fe^2+^ levels were measured using a FerroOrange fluorescent probe (F374; Dojindo) according to the manufacturer’s instructions. Ishikawa cells were inoculated in serum-free medium on μ-slide 8-chambered polymer coverslips (Ibidi, GmbH, Germany) and cultured overnight at 37°C in a 5% CO_2_ incubator. The supernatant was removed, and the cells were washed thrice in serum-free medium. Subsequently, the cells were treated with si-*CAPG*, NC, erastin, or si-*CAPG*+ferrostatin-1 and cultured for 24 h. After washing with serum-free medium, Ishikawa cells were incubated with 1 μM FerroOrange working solution for 30min at 37°C and observed under a fluorescence microscope. Fe^2+^ fluorescence intensity was quantified using ImageJ software.

### Statistical analysis

SPSS software (version 21.0, SPSS, Inc., Chicago, IL, USA) was used for statistical analysis. Univariate and multivariate Cox regression analyses were performed to evaluate the effects of the clinical variables on survival. Comparisons and correlations between the two groups were analyzed using Student’s *t*-test and Pearson’s correlation (normally distributed data) or the non-parametric Mann–Whitney *U* test and Spearman’s correlation (non-normally distributed data). Data were expressed as the mean ± SEM (standard error of mean) of three independent experiments. Statistical significance was set at *P*-value < 0.05 (two-tailed).

## Results

### Elevated expression of *CAPG* in UCEC

**Figure 1:**
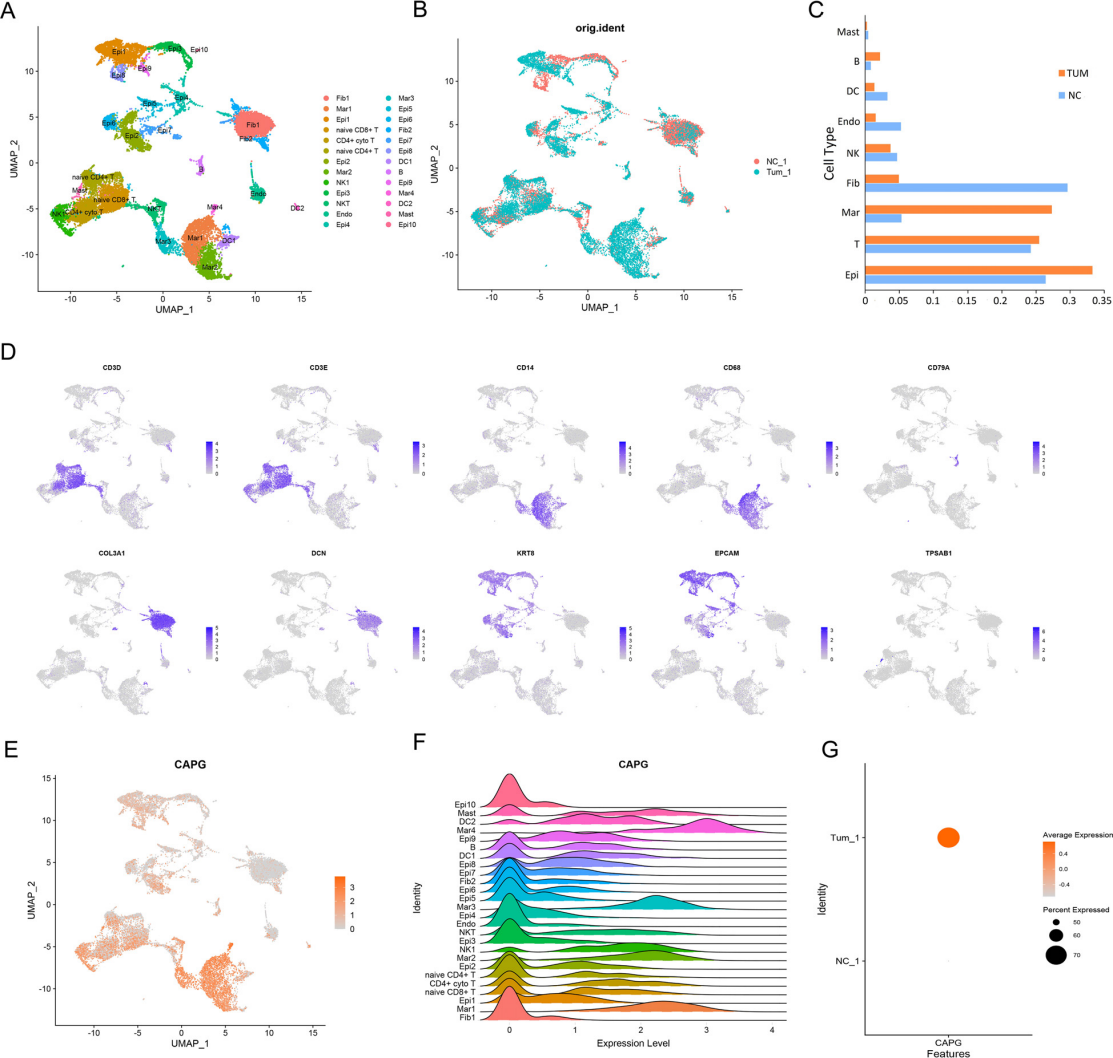
An atlas of immune cells in patients with UCEC. **(A)**, **(B)** A UMAP projection of all cells from one patient with UCEC and one matched healthy control. Different colors indicate cell clusters. **(C)** Box plot highlights the contribution of two groups to each cell cluster. **(D)** Expression of marker genes of each cell type defined in **(A)**. **(E, F)** scRNA-seq analysis of *CAPG* expression in 26 cell subsets. **(G)** Average expression level and percent expression of *CAPG* in tumor and control groups. UMAP: Uniform Manifold Approximation and Projection; scRNA-seq: single-cell RNA-sequencing.

First, single-cell RNA-sequencing (scRNA-seq) data from five UCEC tumor tissues and three para-tumor tissues were merged for systematic comparison across patients and principal component analysis, which revealed 26 cell subsets in the tumor datasets, such as fibroblasts and macrophages (**Figures 1A, B**). The tumor group contributed more in epithelial cells, T cells and macrophages, whereas the control group contributed more in epithelial cells, T cells and fibroblasts (**Figure 1C**). Ten marker genes (*CD3D, CD3E, CD14, CD68, CD79A, COL3A1, DCN, KRT8, EPCAM*, and *TPSAB1*) showed high expression levels in different cell types (**Figure 1D**). The expression level of *CAPG* was relatively high in macrophages (Mar1, Mar2, Mar3, and Mar4) and other cell subsets (**Figures 1E, F**), and *CAPG* was generally highly expressed in the tumor group (**Figure 1G**).

**Figure 2:**
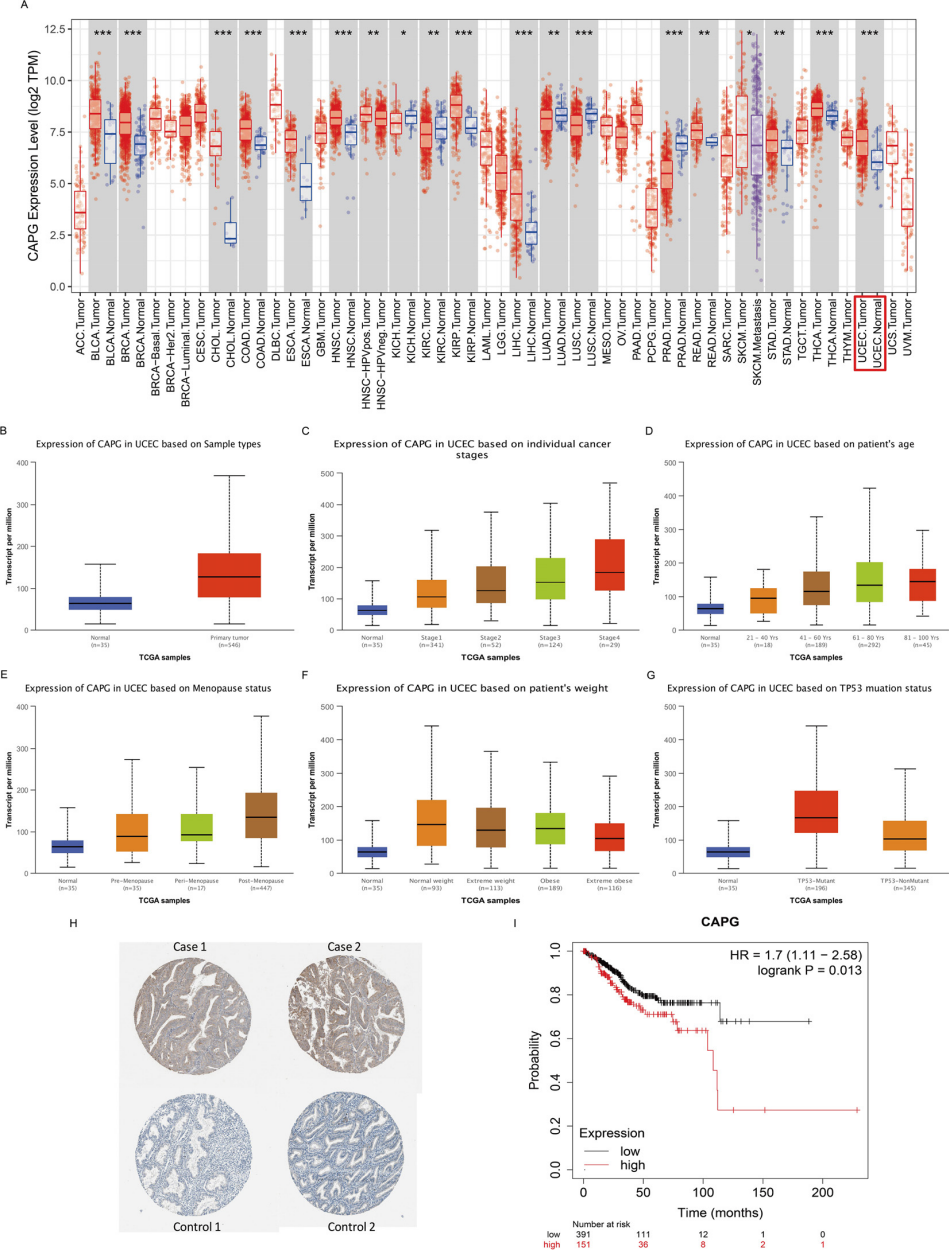
Expression levels of *CAPG* and its correlation with UCEC prognosis. **(A)** Human CAPG expression levels in different cancer types from TCGA data in TIMER. **(B-G)** *CAPG* expression levels in subgroups of UCEC patients by the UALCAN database. **(H)** Immunohistochemical images of CAPG protein in endometrial carcinoma and adjacent tissues. **(I)** Correlation between *CAPG* gene expression and UCEC prognosis. *P < 0.05, **P < 0.01, ***P < 0.001.

Second, we analyzed the expression levels of *CAPG* in different cancer types from TCGA data in TIMER and found that *CAPG* expression level in the UCEC tumor group was significantly higher than that in the corresponding normal group (*P* = 1.98E-07) (**Figure 2A**), which is consistent with the results of the scRNA-seq analysis. Specifically, the expression level of *CAPG* was higher in UCEC primary tumors than in the normal group, and its expression level increased with an increase in individual cancer stage, patient age, and menopausal status but decreased with an increase in patient weight and was higher in the TP53 mutant group than in the non-mutant group (**Figures 2B-G**). In addition, CAPG protein expression was upregulated in endometrial cancer tissues compared to that in normal tissues (**Figure 2H**).

### Prognostic potential of *CAPG* in UCEC

The OS rate of the high CAPG expression group was significantly lower than that of the low *CAPG* expression group (n = 543, log-rank *P* = 0.013, hazard ratio (HR) = 1.7) (**Figure 2I**). Our analysis identified age, stage, and grade as independent prognostic indicators for OS in UCEC (**Table 1**).

**Table 1:** Univariate and multivariate analyses of the correlation between CAPG expression and overall survival rate among UCEC patients.

Parameter	Univariate Analysis		Multivariate analysis	
	HR [95% CI]	P value	HR [95% CI]	P value
Age	1.037 (1.016-1.058)	0.000443	1.041 (1.018-1.065)	0.000355
Stage	1.936 (1.607-2.333)	3.57E-12	1.757 (1.452-2.126)	6.73E-09
Grade	2.728 (1.797-4.141)	0.000002	2.022 (1.325-3.084)	0.001086

### Validation of *CAPG* promoter hypomethylation

**Figure 3:**
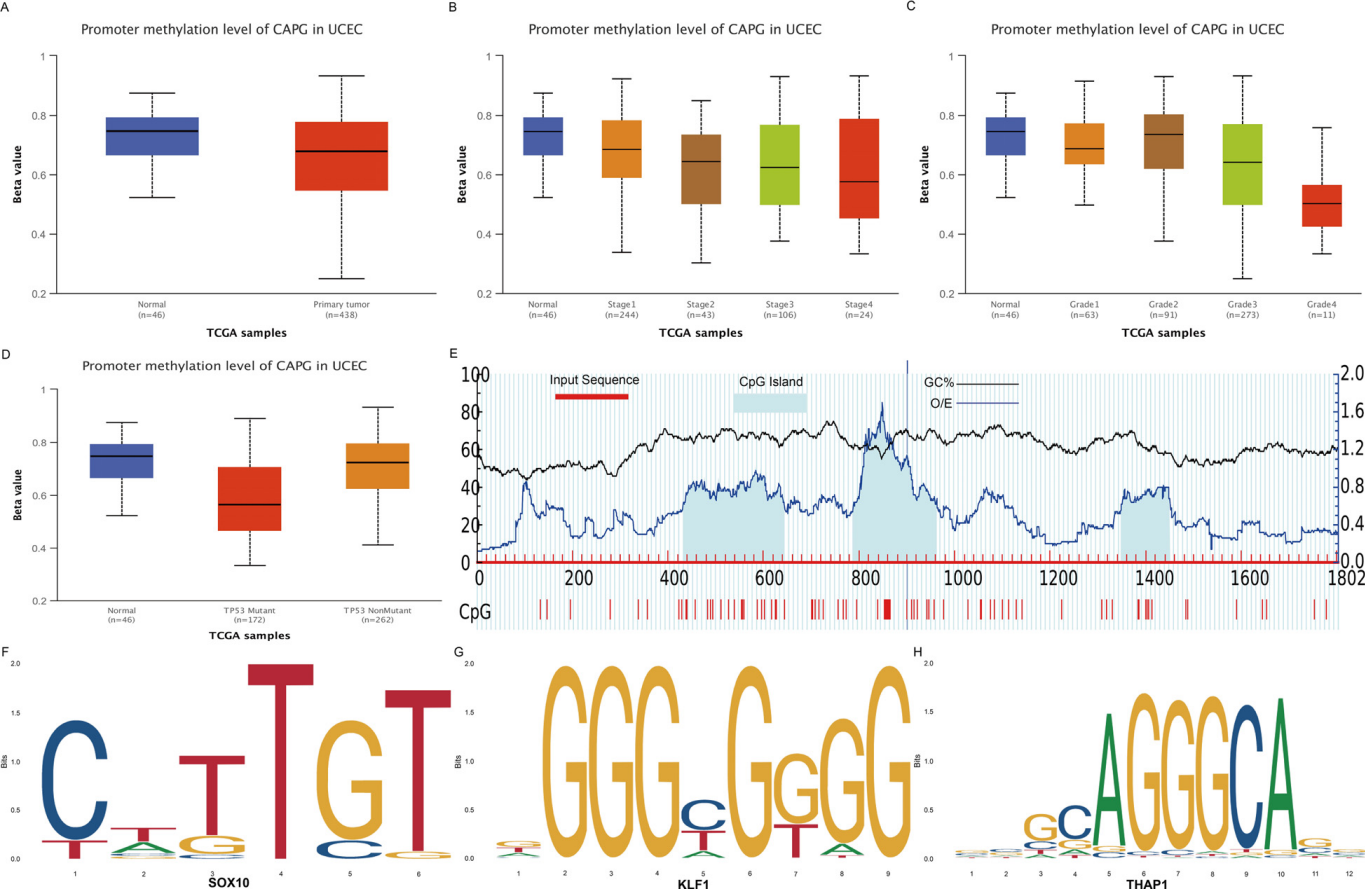
DNA methylation analysis of CAPG gene. **(A)** Methylation levels of CAPG promoters in UCEC and control groups. CAPG promoter methylation levels at different stages **(B)**, grades **(C)**, and TP53 mutant **(D)** of UCEC. **(E)** Distribution of CpG island in the CAPG promoter region. Prediction of transcription factors binding to each CpG island of CAPG: **(F)** SOX10, **(G)** KLF1, **(H)** THAP1.

DNA methylation is a crucial epigenetic modification that is a vital regulator of gene transcription and has carcinogenic effects (27). The *CAPG* promoter methylation level in the primary tumor group was lower than in the normal group (**Figure 3A**). Further subgroup analyses with respect to tumor stage, grade, and TP53 mutation status showed that the methylation level of the *CAPG* promoter decreased with higher tumor stages and was lowest in tumor grade 4, while the TP53 mutant group exhibited lower methylation levels than the TP53 non-mutant group (**Figures 3B-D**). We obtained the DNA promoter sequences of *CAPG* from an online database and analyzed the CpG islands in the promoter. Three CpG islands were identified (**Figure 3E**), and each island contained multiple transcription factors (TF), which were ranked according to their relative scores. The top five TFs are shown in **Supplementary Table S2**, among which *SOX10*, *KLF1*, and *THAP1* were most closely associated with a single island (**Figures 3F-H**).

### Identification of miRNAs targeting *CAPG*

To explore the regulators of *CAPG* associated with UCEC, we further analyzed miRNAs potentially targeting *CAPG* from the TargetScan and TarBase v.8 databases and the top 100 miRNAs in the miRWalk database. Notably, the negative correlation between miRNA and target gene expression indicated that higher-grade UCEC exhibit lower miRNA expression. Therefore, we identified hsa-miR-497-5p and hsa-miR-424-5p, whose expression levels were significantly lower in the tumor group compared to the normal group (**Supplementary Figures S1A, B**), and the pronounced difference in OS rates further affirmed the prognostic value of these miRNAs, as the low expression group exhibited reduced OS rates (**Supplementary Figures S1C, D**).

### Correlation and enrichment analyses

**Figure 4:**
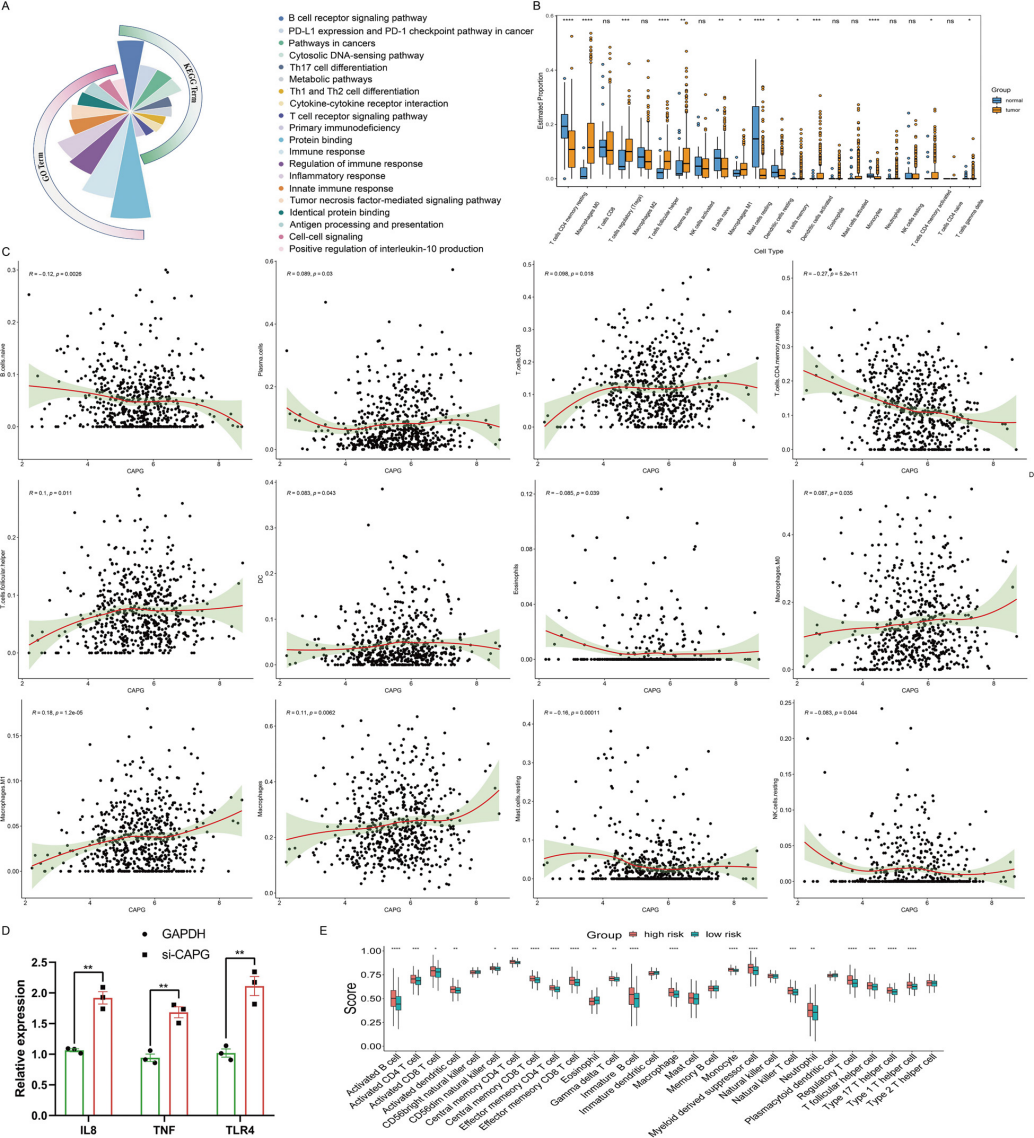
Analysis of immune cell infiltration in UCEC. (A) GO and KEGG analyses of CAPG in UCEC. (B) Estimated proportion of each-type immune cell in tumor and normal endometrium tissues using CIBERSORT algorithm. (C) Scatterplot of correlation between CAPG expression in UCEC and immune infiltration level. (D) Relative expression levels of immune-related genes IL8, TNF, and TLR4 in Ishikawa cells treated with si-CAPG. (E) Comparison of ssGSEA scores of the 28-types immune cells between high- and low-risk group. *P < 0.05, **P < 0.01, ***P < 0.001, ****P < 0.0001, ns: no significant (P > 0.05).

To elucidate functional repertoire of *CAPG* and its implications in pertinent pathways, we conducted a correlation analysis between *CAPG* and 300 genes in UCEC using TCGA data (**Supplementary Table S3**), and found that *CAPG* was primarily associated with immune-related gene terms, including regulation of immune response, inflammatory response, tumor necrosis factor-mediated signaling pathway, and positive regulation of interleukin (IL)-10 production. In addition, KEGG analysis indicated enrichment and crosstalk of these genes in the B cell receptor signaling pathway, PD-L1 expression, PD-1 checkpoint pathway, Th1 and Th2 cell differentiation, and the T cell receptor signaling pathway (**Figure 4A**). These findings indicate that *CAPG* is linked to numerous pathways related to malignancy in UCEC, particularly the pathways associated with the immune system.

### Correlation between *CAPG* expression and immune cell infiltration

Tumor-infiltrating immune cells affect the survival of patients with various cancers. In UCEC, there were significant differences in the estimated proportions of resting CD4 memory T cells, M0 macrophages, regulatory T cells (Tregs), follicular helper T cells, plasma cells, naive B cells, M1 macrophages, resting mast cells, resting dendritic cells, memory B cells, activated dendritic cells, monocytes, activated CD4 memory T cells, and gamma delta T cells between the tumor and normal groups (**Figure 4B**). CAPG expression level displayed a significant positive correlation with the infiltration level of plasma cells (*R* = 0.089, *P* = 0.03), CD8 T cell (*R* = 0.098, *P* = 0.018), follicular helper T cells (*R* = 0.1, *P* = 0.011), DC (*R* = 0.083, *P* = 0.043), M0 macrophages (*R* = 0.087, *P* = 0.035), M1 macrophages (*R* = 0.18, *P* = 1.2e-05), and macrophages (*R* = 0.11, *P* = 0.0062), and negative correlation with naive B cells (*R* = -0.12, *P* = 0.0026), resting CD4 memory T cells (*R* = -0.27, *P* = 5.2e-11), eosinophils (*R* = -0.085, *P* = 0.039), resting mast cells (*R* = -0.16, *P* = 0.00011), and resting natural killer (NK) cells (*R* = -0.083, *P* = 0.044) (**Figure 4C**). Moreover, the correlation analysis between CAPG and gene markers of infiltrating immune cells in TIMER, including B cell, CD8+ T cell, Tfh, Th1, Th2, Th9, Th17, Th22, Treg, T cell exhaustion, macrophage, M1, M2, TAM, monocyte, neutrophil, NK cell, and dendritic cell, reinforced this intricate relationship, and we found some significant related gene markers, such as CD19, CD11b, CD11c (**Table 2**). Additionally, immune-related genes IL8, TNF, and TLR4 were significantly highly expressed after CAPG knockdown (*P* < 0.01) (**Figure 4D**).

**Table 2:** Correlations between CAPG and gene markers of immune cells in UCEC by TIMER (None and Purity) or by Pearson analysis (Tum our).

Cell type	Gene marker	None		Purity		Tum our	
		Cor	*P*	Cor	*P*	R	*P*
B cell	CD19	0.146	**6.40E-04**	0.176	**2.57E-03**	0.25	**7.31E-10**
	CD79A	0.172	**5.42E-05**	0.162	**5.40E-03**	0.21	**2.83E-07**
	CD79B	0.142	**9.21E-04**	0.088	1.32E-01	0.283	**2.63E-12**
	CD20 (KRT20)	0.022	6.04E-01	0.038	5.15E-01	0.07	0.088
	CD38	0.256	**1.41E-09**	0.224	**1.13E-04**	0.261	**1.26E-10**
CD8+ T cell	CD8A	0.121	**4.71E-03**	0.106	7.09E-02	0.277	**8.41E-12**
	CD8B	0.048	2.64E-01	-0.01	8.59E-01	0.084	**0.041279**
Tfh	BCL6	-0.051	2.32E-01	-0.078	1.81E-01	-0.132	**0.001365**
	ICOS	0.104	**1.52E-02**	0.114	5.04E-02	0.239	**4.31E-09**
	CXCR5	0.168	**8.01E-05**	0.165	**4.58E-03**	0.142	**0.000542**
Th1	T-bet (TBX21)	0.103	**1.63E-02**	0.092	1.17E-01	0.353	**1.03E-18**
	STAT4	-0.027	5.27E-01	-0.041	4.84E-01	0.086	**0.037**
	IL12RB2	0.28	**2.71E-11**	0.279	**1.26E-06**	0.173	**0.036596**
	WSX1 (IL27RA)	0.173	**5.25E-05**	0.196	**7.65E-04**	0.224	**0.000024**
	STAT1	0.353	**1.61E-17**	0.355	**4.20E-10**	0.27	**3.69E-08**
	IFN-γ (IFNG)	0.133	**1.80E-03**	0.144	**1.38E-02**	0.294	**3.12E-13**
	TNF-α (TNF)	0.289	**7.64E-12**	0.382	**1.29E-11**	0.13	**0.00157**
Th2	GATA3	0.205	**1.43E-06**	0.214	**2.17E-04**	0.023	0.578
	CCR3	0.081	5.89E-02	0.038	5.18E-01	-0.021	0.618
	STAT6	0.097	**2.35E-02**	0.146	**1.26E-02**	-0.009	0.826
	STAT5A	0.301	**7.58E-13**	0.312	**4.79E-08**	0.357	**4.22E-19**
Th9	TGFBR2	0.16	**1.74E-04**	0.159	**6.36E-03**	-0.109	**0.007897**
	IRF4	0.119	**5.51E-03**	0.108	6.60E-02	0.21	**2.75E-07**
	PU.1 (SPI1)	0.276	**6.22E-11**	0.296	**2.38E-07**	0.427	**1.65E-27**
Th17	STAT3	0.031	4.65E-01	0.087	1.38E-01	-0.084	**0.04089**
	IL-21R	0.197	**3.80E-06**	0.196	**7.42E-04**	0.371	**1.14E-20**
	IL-23R	-0.022	6.09E-01	-0.037	5.33E-01	-0.055	0.18
	IL-17A	0.059	1.66E-01	0.098	9.29E-02	0.006	0.877
Th22	CCR10	0.125	**3.45E-03**	0.109	6.23E-02	0.123	**0.00286**
	AHR	-0.009	8.35E-01	-0.037	5.24E-01	-0.104	**0.011375**
Treg	FOXP3	0.083	5.15E-02	0.099	9.13E-02	0.181	**0.00001**
	CD25 (IL2RA)	0.108	**1.18E-02**	0.084	1.53E-01	0.318	**2.86E-15**
	CCR8	0.042	3.24E-01	0.094	1.10E-01	0.046	0.263
T cell exhaustion	PD-1 (PDCD1)	0.118	**5.70E-03**	0.054	3.55E-01	0.268	**3.76E-11**
	CTLA4	0.006	8.85E-01	-0.028	6.29E-01	0.123	**0.002828**
	LAG3	0.212	**6.15E-07**	0.196	**7.60E-04**	0.334	**7.60E-17**
	TIM-3 (HAVCR2)	0.269	**2.05E-10**	0.261	**5.81E-06**	0.41	**2.61E-25**
Macrophage	CD68	0.336	**9.70E-16**	0.355	**4.19E-09**	0.098	**0.017359**
	CD11b (ITGAM)	0.206	**1.22E-06**	0.195	**8.10E-04**	0.305	**4.02E-14**
M1	INOS (NOS2)	0.093	**2.91E-02**	0.145	**1.30E-02**	0.123	**0.002686**
	IRF5	0.438	**<9.70E-16**	0.466	**3.34E-17**	0.426	**2.03E-27**
	COX2 (PTGS2)	-0.117	**6.22E-03**	-0.172	**3.18E-03**	-0.049	0.232
M2	FCGR3A	0.221	**2.09E-07**	0.16	**5.97E-03**	0.342	**1.24E-17**
	ARG1	-0.048	2.59E-01	-0.069	2.40E-01	-0.059	0.151
	MRC1	0.024	5.78E-01	0.015	8.05E-01	0.228	**2.10E-08**
	MS4A4A	0.218	**2.98E-07**	0.176	**2.50E-03**	0.304	**4.49E-14**
TAM	CCL2	0.04	3.53E-01	0.015	8.00E-01	-0.011	0.786
	CD80	0.252	**2.32E-09**	0.266	**3.90E-06**	0.268	**4.06E-11**
	HLA-G	0.026	5.44E-01	0.057	3.29E-01	0.147	**0.00034**
	CD86	0.265	**3.45E-10**	0.278	**1.36E-06**	0.381	**8.47E-22**
	CCR5	0.202	**2.13E-06**	0.195	**7.99E-04**	0.373	**7.28E-21**
Monocyte	CD14	0.316	**5.35E-14**	0.311	**5.68E-08**	0.455	**7.28E-21**
	FCGR3B	0.038	3.79E-01	0.068	2.43E-01	-0.017	0.679
	CD115 (CSF1R)	0.239	**1.72E-08**	0.236	**4.58E-05**	0.354	**8.77E-19**
Neutrophil	CD66b (CEACAM8)	-0.106	**1.37E-02**	-0.142	**1.52E-02**	-0.045	0.276
	MPO	0.021	6.21E-01	0.011	8.57E-01	0.017	0.688
	CD15 (FUT4)	0.242	**1.06E-08**	0.234	**5.39E-05**	0.099	**0.016015**
	CD11b (ITGAM)	0.206	**1.22E-06**	0.195	**8.10E-04**	0.305	**4.02E-14**
Natural killer cell	XCL1	0.119	**5.37E-03**	0.175	**2.65E-03**	0.054	0.187
	CD7	0.204	**1.74E-06**	0.172	**3.19E-03**	0.343	**1.09E-17**
	KIR3DL1	0.109	**1.08E-02**	0.111	5.72E-02	0.02	0.633
Dendritic cell	CD1C (BDCA-1)	0.058	1.79E-01	0.064	2.77E-01	0.099	**0.016203**
	CD141 (THBD)	-0.067	1.18E-01	-0.138	**1.85E-02**	-0.118	**0.003978**
	CD11c (ITGAX)	0.24	**1.40E-08**	0.24	**3.19E-05**	0.306	**2.82E-14**

Tfh, follicular helper T cell; Th, T helper cell; Treg, regulatory T cell; TAM, Tumor-associated macrophage. None: Correlation without adjustment. Purity: Correlation adjusted by purity. Cor, R value of Spearman’s correlation. Bold values indicate a significant correlation between CAPG and gene markers of immune cells in UCEC.

### Correlation between *CAPG* expression and tumor microenvironment

Capitalizing on the immune/stromal/ESTIMATE/tumor purity scores, our assessment of UCEC cases within the low- and high-risk groups revealed stark contrasts. The low-risk group exhibited lower Immune and ESTIMATE scores, coupled with a notably higher tumor purity score (*P <*0.0001) but no significant difference in stromal score (*P >*0.05). Furthermore, the high-risk group had more robust ssGSEA scores across numerous immune cell types, indicating increased immune cell infiltration (**Figure 4E**). These insights collectively emphasized the intrinsic relationship between *CAPG* expression and the complex tumor microenvironment.

### Ferroptosis analysis

**Figure 5:**
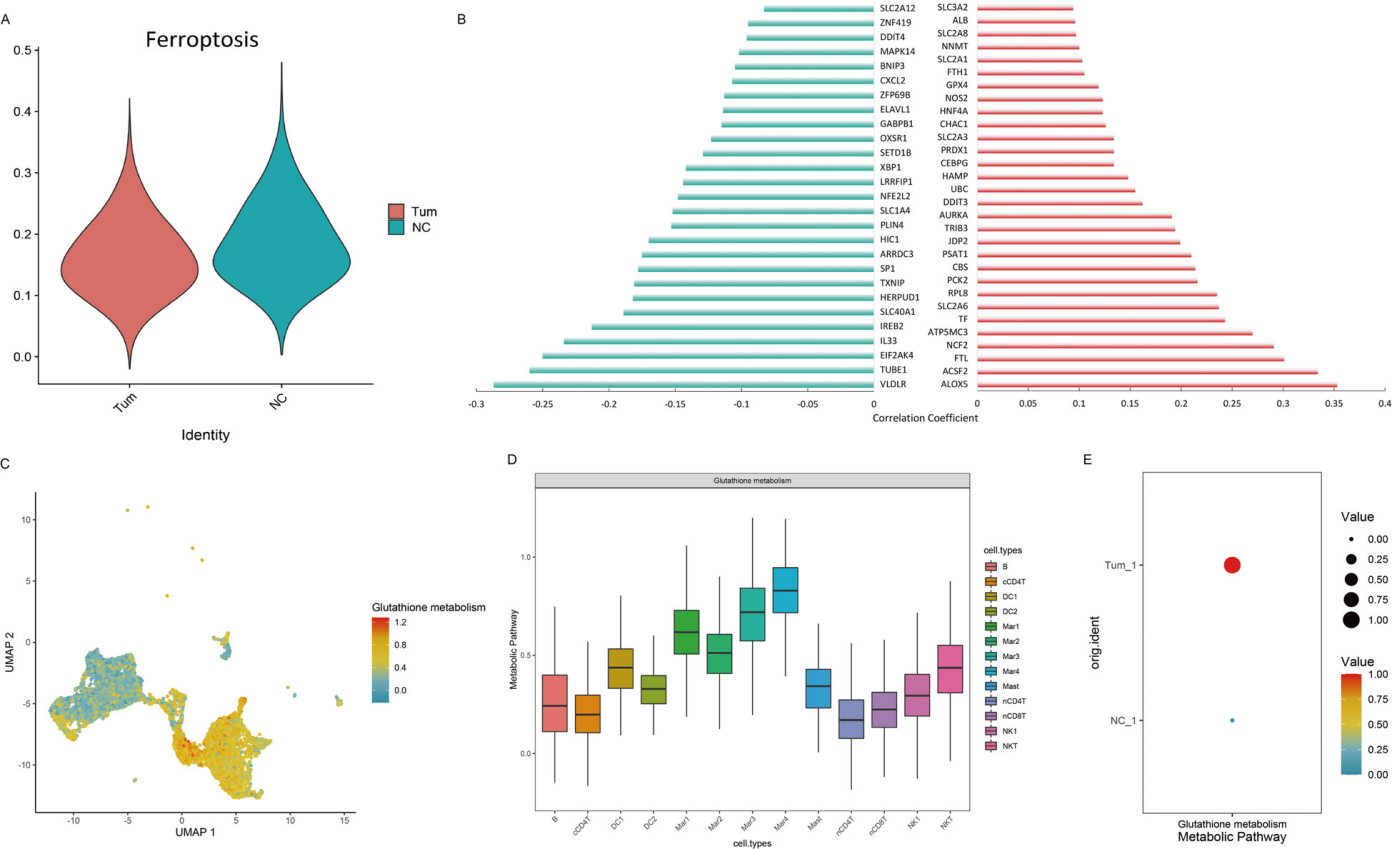
Ferroptosis analysis in UCEC. **(A)** Ferroptosis scores in scRNA-seq analysis. **(B)** Correlation between *CAPG* expression level and ferroptosis marker set. Left: negative correlation; Right: positive correlation. **(C, D)** Metabolic scores of ferroptosis-related metabolic pathways in UCEC immune cells. **(E)** Glutathione metabolism level in tumor and control groups.

We performed ferroptosis analysis at the scRNA-seq level and found that the ferroptosis scores were lower in the tumor group than in the normal group (**Figure 5A**). This prompted a comprehensive analysis of the correlation between *CAPG* and ferroptosis markers, elucidating intriguing links, particularly with *ALOX5* and *VLDLR*, which were most closely related to *CAPG* among the markers (**Figure 5B**). Furthermore, we dissected the metabolic pathways related to ferroptosis (28–30), revealing metabolic discrepancies within the tumor tissues. As a metabolic pathway closely related to ferroptosis, the metabolic scores of glutathione metabolism were analyzed, revealing that UCEC immune cells (such as Mar, DC, B, and mast cells) had relatively high ferroptosis scores (**Figures 5C, D**). The level of glutathione metabolism in the tumor group was generally higher than that in the control group (**Figure 5E**). These findings highlighted the intricate role of *CAPG* in ferroptosis. Additionally, *CAPG*-related drugs were analyzed, and decitabine was identified as an inducer of ferroptosis.

### Potential chemotherapeutics for UCEC patients

Our investigation was extended to identify of potential therapeutic avenues for UCEC based on the *CAPG* expression profile. Intriguingly, paclitaxel (*P* = 8.83E-03), epirubicin (*P* = 2.36E-04), docetaxel (*P* = 1.28E-02), and cyclophosphamide (*P* = 1.46E-07) exhibited significantly higher IC50 values in the high-risk cohort than that in the low-risk cohort, whereas for gemcitabine, topotecan, and cisplatin (*P* > 0.05), no significant differences were observed between the low- and high-risk cohorts (**Supplementary Figures S2A-G**), indicating that paclitaxel, epirubicin, docetaxel, and cyclophosphamide were more suitable for UCEC patients in the low *CAPG* expression cohort.

### Potential physiological function of *CAPG* on Ishikawa cells

**Figure 6:**
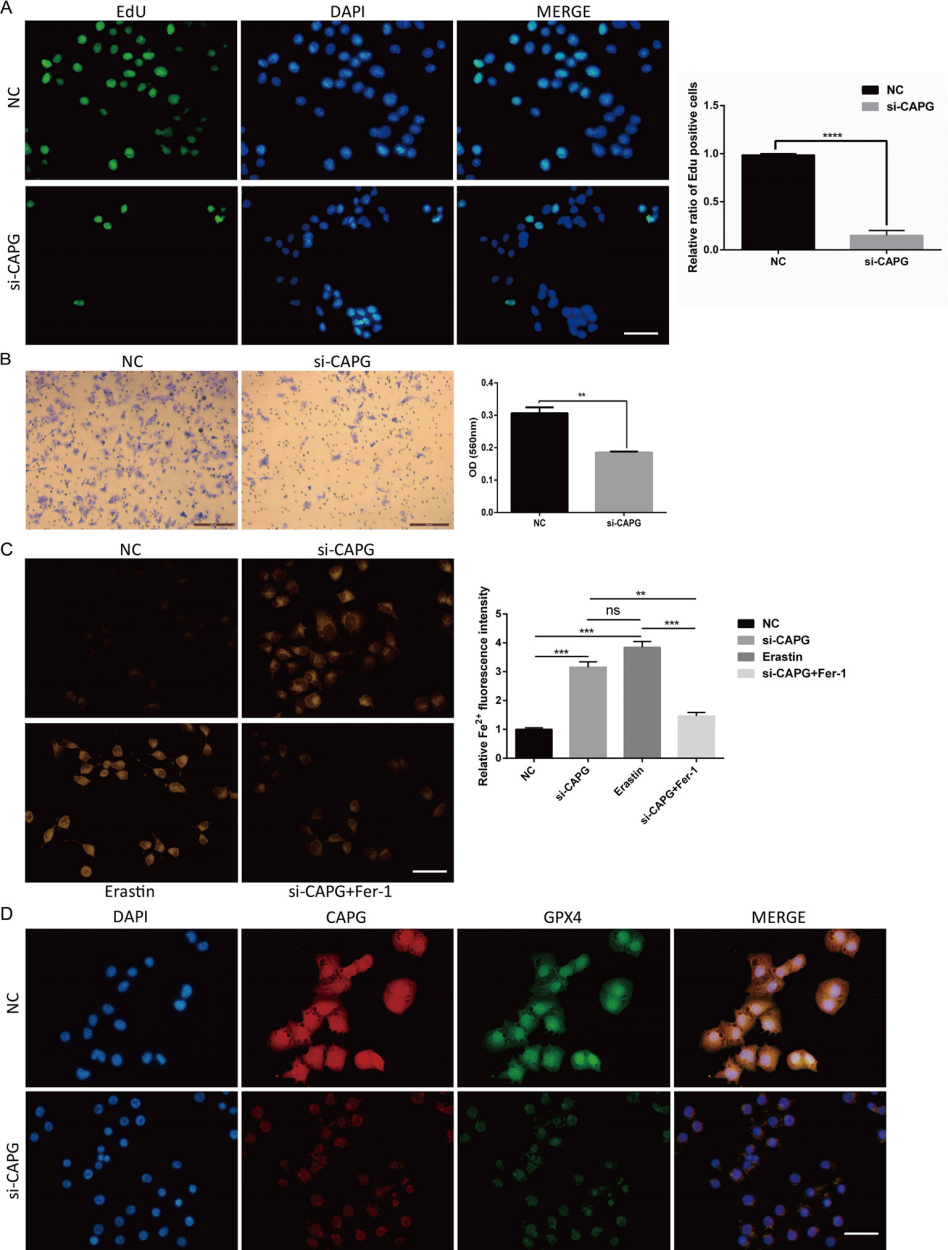
Potential physiological function of *CAPG* on Ishikawa cells, si-*CAPG*-induced ferroptosis, and the expression and co-localization of *CAPG* and *GPX4* in Ishikawa cells. **(A)** EdU staining demonstrated that si-*CAPG* significantly inhibited the proliferation of Ishikawa cells. Scale bar = 40μm. **(B)** Invasion of Ishikawa cells treated with si-*CAPG* was evaluated by transwell assay. Scale bar = 200μm. **(C)** Representative images of each group of Ishikawa cells treated by NC, si-*CAPG*, erastin and si-*CAPG*+Fer-1 and stained by FerroOrange were captured. Scale bar = 40μm. **(D)** Representative fluorescence images of nuclear and cytoplasmic localization of CAPG (red) and GPX4 (green) in UCEC cell line by immunofluorescence. Nucleus was stained with DAPI (blue). Scale bar = 40μm. **P < 0.01, ***P < 0.001, ****P < 0.0001, ns: no significant (*P* > 0.05). DAPI: 4′,6-diamidino-2-phenylindole.

To elucidate the physiological function of *CAPG* on Human UCEC cell line Ishikawa cells, we designed and synthesized *CAPG* knockdown sequences from Santa Cruz Biotechnology (code number: sc-44920). Notably, our functional assays involving EdU staining and Transwell migration assays illustrated pronounced suppression of cell proliferation and invasion upon *CAPG* knockdown (**Figures 6A, B**).

### si-*CAPG*−induced ferroptosis

FerroOrange staining showed that si-*CAPG* markedly increased intracellular Fe^2+^ levels in Ishikawa cells (**Figure 6C**). *GPX4* is a key target of ferroptosis. Immunofluorescence analysis revealed that CAPG and GPX4 were colocalized in the nucleus of Ishikawa cells and confirmed the downregulation of CAPG and GPX4 proteins after *CAPG* knockdown (**Figure 6D**), suggesting that *CAPG* may regulate and coordinate with *GPX4* to further affect ferroptosis.

## Discussion

This study presents a comprehensive analysis of potential of *CAPG* as an immunogenic and prognostic biomarker for UCEC. Our investigation delved into the intricate interplay between *CAPG* expression, DNA methylation, miRNA regulation, ferroptosis, and the TME. Notably, we found that abnormal methylation levels of *CAPG* and expression of *CAPG*-related miRNAs may trigger changes in *CAPG* expression, consequently impacting immune response, ferroptosis, and patient prognosis. Furthermore, our experimental evidence suggests that *CAPG* is involved in UCEC initiation and progression by influencing cell proliferation, invasion, and ferroptosis. It is plausible that ferroptosis leads to changes in the TME, which in turn leads to cellular metabolic disorders, resulting in poor prognosis in patients with advanced UCEC.

DNA methylation and miRNAs have been extensively studied as modes of epigenetic modification in human tumors. Aberrant DNA methylation has been linked to changes in gene expression during the progression of many tumor types, including UCEC (31). The promoter of *CAPG* is regulated by DNA methylation and is involved in integrin α6β4 regulating the invasion and metastasis of breast cancer cells (32). Similarly, our study confirmed that methylation level changes in the *CAPG* promoter region may alter its expression level to induce UCEC. We found that these changes were intricately associated with tumor stage, grade, TP53 mutation status, and TFs on different CpG islands. In parallel, miRNAs targeting *CAPG* genes have also emerged as crucial regulators in UCEC; hsa-miR-497-5p, a sponge of hsa_circ_0011324, targets mTOR and participates in the progression of UCEC (33). Abnormal expression of has-miR-424-5p enhances the proliferation and invasion of tumor cells by targeting LATS1 gene (34). These results, combined with those of our study, suggest that the abnormal expression of *CAPG* may induce UCEC by altering cell proliferation and invasion owing to DNA methylation or miRNA changes. However, the interactions between DNA methylation, miRNA, and *CAPG* and the detailed mechanisms need to be further verified.

Correlation and enrichment analyses underscored the centrality of *CAPG* in immune regulation and its involvement in many malignancy-related pathways in UCEC. Immune cell infiltration and TME play important roles in the physiological and pathological processes of tumorigenesis (35, 36). From another perspective, improving immunogenicity by altering the TME may be key to achieving precise personalized cancer treatment in the future (37).

The presence of immune cells in TME has a significant impact on tumor growth, drug resistance, and epithelial-mesenchymal transformation (38). Various infiltrating immune cells, including neutrophils, macrophages, Treg cells, helper T cell 2, tumor-associated fibroblasts, and bone marrow-derived suppressor cells, are key factors in mediating the immunosuppressive microenvironment. Neutrophils can promote tumor progression by stimulating tumor cell proliferation, promoting angiogenesis, tumor metastasis, and degrading the matrix (39). Tumor-associated fibroblasts can release a variety of chemokines and cytokines, such as *IL-6* and *CXCL2*, to promote the migration and aggregation of Tregs cells into the TME, thereby transforming TME into an immunosuppressor (40). Th1 cells play a crucial role in the formation of anti-tumor immune responses, while Th2 cells can inhibit the anti-tumor immune effect in the body by secreting a variety of cytokines. Additionally, Th9 cell differentiation plays a dual role in tumor development (41). We speculate that CAPG may affect the TME by changing the function of these infiltrating immune cells and participate in the occurrence and development of UCEC. Further, our investigation indicated that the extent of *CAPG* expression was pronouncedly positively associated with the infiltration levels of plasma cells, CD8 T cells, follicular helper T cells, M0 macrophages, M1 macrophages, and macrophages and negatively associated with naive B cells, resting CD4 memory T cells, eosinophils, resting mast cells, and resting NK cells. The potential correlation between CAPG expression and the gene markers of infiltrating immune cells in TIMER was further analyzed, such as *CD19*, *CD11b*, *CD11c*. Immune, tumor purity, and ESTIMATE scores were significantly correlated with *CAPG* levels, and the level of immune cell infiltration in the high-risk group was stronger than that in the low-risk group.

Moreover, IL8, TNF and TLR4, as common immunomodulators, are significantly highly expressed after CAPG knockdown, suggesting that IL8, TNF and TLR4 signaling may be one of the mechanisms of CAPG regulating immune cell infiltration. IL-8 is secreted by a variety of immune cells, such as macrophages, neutrophils, and T lymphocytes. IL8 has been reported to promote cancer progression and metastasis mainly through its ability to attract and functionally regulate neutrophils and macrophages, not only recruiting neutrophils to tumor lesions, but also triggering the extrusion of neutrophil extracellular traps (42), which is very important for the regulation of tumor microenvironment and anti-tumor immunotherapy (43). TNF is mainly secreted by macrophages and is a gene marker of Th2 cells, suggesting a role for Th2 cells in UCEC in this study. It has also been reported that INF expression is associated with immune cell infiltration and may serve as an immune-marker gene in UCEC immunotherapy (44). Furthermore, studies have also shown that TLR4 ligands serve as potent immune adjuvants in aggressive malignancies, and TLR4 in most tumor cells (including UCEC) can change the tumor microenvironment to escape immune surveillance, while activated TLR4 can also enhance the immune response and produce anti-tumor effects (45). TLR4 mediates the secretion of IFN Iα and participates in the immune escape of UCEC (46). The expression of TLR4 is closely related to the infiltration of macrophages, T cells and B cells (47). TLR4 on the surface of macrophages interacts with CCRL2 to promote anti-tumor T cell immunity by enhancing TLR4-mediated immune-stimulated macrophage activation (48). Activation of dendritic cells by TLR4 plays an important role in enhancing anti-tumor T cell immune response (49). Taken together, these findings indicate that changes in *CAPG* expression affect the normal function of immune cell infiltration and the TME and play a crucial role in the regulation of immune responses in UCEC.

Glutathione (GSH) peroxidase 4 (*GPX4*) is a major detoxifying enzyme that uses GSH as a substrate to reduce lethal lipid peroxides that can induce cell death; therefore, inactivation of *GPX4* increases lipid peroxides, thereby promoting the ferroptosis process (14). It has been shown that CAPG regulates ferroptosis through SLC7A11-mediated GSH synthesis to promote cell proliferation, and the GSH level is decreased after CAPG knockdown in hepatocellular carcinoma (21). In the TME, anti-tumor immune cells are highly sensitive to ferroptosis, and *GPX4* exerts protective effects on T and B cells. Tregs resist ferroptosis by upregulating *GPX4* expression. Under high-lipid conditions, CD8+ T cells increase the uptake and storage of cholesterol and fatty acids by upregulating the expression of *CD36*, whereas *CD36* overexpression can induce lipid peroxidation and trigger ferroptosis in CD8+ T cells (50). CD8+ T cells secrete IFN-γ and undergo lipid peroxidation through *ACSL4*, thereby regulating the sensitivity of ferroptosis. IFN-γ can upregulate the expression of *PD-L1* on the surface of endometrial cancer cells, thereby promoting their growth. Taken together, inducing ferroptosis in the TME to restrict tumors leads to the death of anti-tumor immune cells, resulting in potential immune escape (50, 51). Additionally, Lv et al. (52) found that an increase in decitabine-induced reactive oxygen species caused myelodysplastic syndrome cell ferroptosis by decreasing GSH levels and *GPX4* activity, which is also consistent with our study. In our study, ferroptosis scores were lower in the tumor group than in the normal group at the scRNA-seq level, and glutathione metabolism levels in the tumor group were generally higher than those in the control group, which may be crucial for clarifying the mechanism of UCEC. Among ferroptosis markers, *ALOX5 (* 53) and *VLDLR (* 54) were the top *CAPG*-related markers whose roles in ferroptosis have been reported. Additionally, the expression level of *CAPG* was positively correlated with that of *GPX4*, and both colocalized in the nucleus of Ishikawa cells, indicating that high expression of *CAPG* and *GPXA* co-regulated ferroptosis, thus promoting the progression of UCEC.

Our exploration of potential chemotherapeutic agents for UCEC patients identified paclitaxel, epirubicin, docetaxel, and cyclophosphamide as promising options, particularly in the low-*CAPG* expression cohort. Paclitaxel is a classic first-line treatment for UCEC and a promising immunotherapeutic agent (55, 56). Paclitaxel may inhibit tumors by modulating several interactions within the TME and by inducing tumor cell ferroptosis (57, 58). Epirubicin, docetaxel, and cyclophosphamide have also been used for the clinical treatment of UCEC (59–62). Among them, low dose cyclophosphamide induces anti-tumor T-cell response (63), modulates tumor microenvironment through TGF-β pathway (64), and induces ferroptosis through Heme Oxygenase-1 (65), which plays an important immunomodulatory role in cancer immunotherapy (66). These results provide a theoretical basis for drug therapy and immunotherapy of UCEC.

Although we integrated information from multiple databases, including single-cell sequencing and methylation modification of *CAPG*, and elucidated the association between *CAPG* and UCEC, this study had some limitations. First, considering that the data was obtained from public resources, systematic biases in the analytical data, such as data heterogeneity and platform differences, cannot be ignored. However, our study is primarily based on a TCGA database (derived from high-throughput sequencing), which may reduce these biases. Second, the number of cohorts with TCGA subjects is limited, and larger sample sizes are required. Third, our findings may prove that *CAPG* expression is associated with immune cell infiltration and ferroptosis in UCEC and patient survival. Nevertheless, how *CAPG* affects patient survival through immune infiltration or ferroptosis remains to be further studied. Finally, the results of this study were derived from bioinformatics analysis and partial experimental validation *in vitro*. Future in-depth mechanistic studies and clinical validation of *CAPG* at the cellular and molecular levels will help clarify how *CAPG* affects immune cell infiltration and ferroptosis *in vitro* and *in vivo*, as well as the clinical effect of immunotherapy on UCEC.

In conclusion, our study shed light on the multifaceted roles of *CAPG* in the pathogenesis of UCEC. These findings highlight the intricate connections between *CAPG* expression, DNA methylation, miRNA regulation, ferroptosis, and the TME. Abnormal expression of *CAPG* in UCEC may alter cell proliferation and invasion owing to DNA methylation or miRNA changes, and high *CAPG* level may participate in tumor progression by influencing the TME to regulate the immune response and ferroptosis. Therefore, *CAPG* is expected to serve as a novel biomarker for identifying patients willing to undergo ferroptosis-induction therapy or combination immunotherapy, which has an important prognostic value in UCEC patients. These data provide implications for immune-based anti-tumor strategies, and valuable insights for the future personalized treatment of UCEC patients.

## Data Availability

The original contributions presented in the study are included in the article/**Supplementary Material**. Further inquiries can be directed to the corresponding author.
