# Supplementary material for: Identification of CAPG as a potential prognostic biomarker associated with immune cell infiltration and ferroptosis in uterine corpus endometrial carcinoma
Source: Front Endocrinol (Lausanne). 2024 Nov 12;15:1452219. doi: 10.3389/fendo.2024.1452219 (PMC11588481; doi:10.3389/fendo.2024.1452219)
Supplement: Supplementary file 1 [file DataSheet1.zip › Supplementary Files/Supplementary Figures.doc]

**Identification of CAPG as a potential prognostic biomarker associated with immune cell infiltration and ferroptosis in uterine corpus endometrial carcinoma**

Junwei Liu^1#^, Weiqiang Zhu^2#^, Lingjin Xia^2#^, Qianxi Zhu^2^, Yanyan Mao^2^, Yupei Shen^2^, Min Li^2^, Zhaofeng Zhang^2^, Jing Du^2^*

#These authors contributed equally to this work.

*Corresponding author: Jing Du. E-mail address: [dujing42@126.com](mailto:dujing42@126.com)

**Supplementary Figure 1** Analysis of miRNAs targeting CAPG gene. (**A, B**) Expression levels of miRNAs targeting CAPG genes in UCEC. (**C, D**) Correlation between expression levels of miRNAs targeting CAPG gene and UCEC prognosis.

**Supplementary Figure 2** Sensitivity analysis of chemotherapeutic drugs for UCEC patients in low- and high-risk cohorts. (**A**) Paclitaxel, (**B**) Epirubicin, (**C**) Docetaxel, (**D**) Cyclophosphamide, (**E**) Gemcitabine, (**F**) Topotecan, and (**G**) Cisplatin. **P* < 0.05, ***P* < 0.01, ****P* < 0.001, *****P* < 0.0001, ns: no significant (*P* > 0.05).
